# Supplementary figures and images for: Pharmacological evidence for the implication of noradrenaline in effort
Source: PLoS Biol. 2020 Oct 12;18(10):e3000793. doi: 10.1371/journal.pbio.3000793 (PMC7580990; doi:10.1371/journal.pbio.3000793)

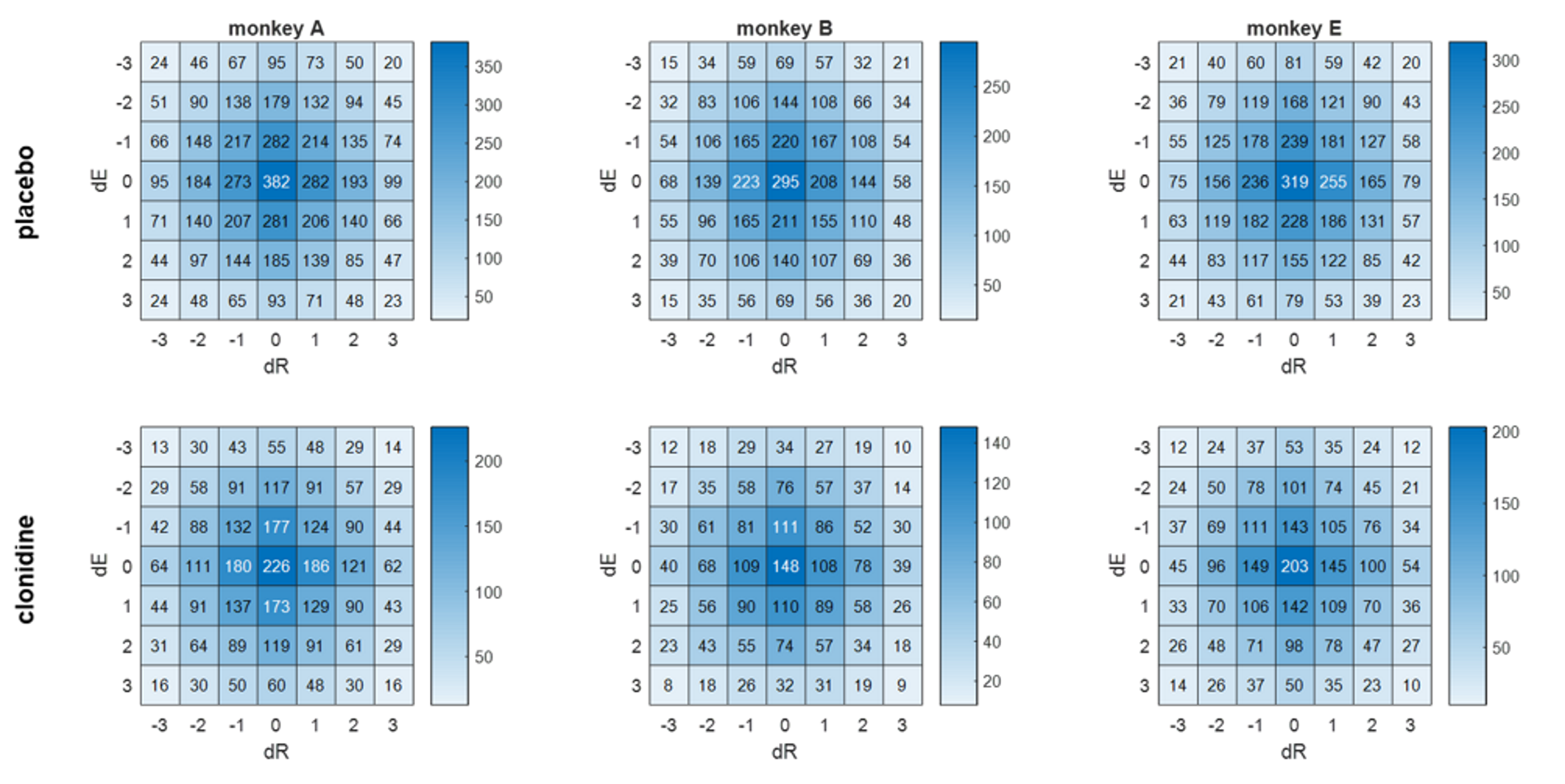

Supplement: S2 Fig — Absolute numbers of trials are indicated inside each box. Note that there is a difference between the placebo and drug session in the absolute numbers (because there were more placebo sessions included) but no difference in the relative proportions. Fig 1. Distribution of experimental conditions across placebo and drug sessions for all monkeys. Absolute numbers of trials are indicated inside each box. Note that there is a difference between the placebo and drug session in the absolute numbers (because there were more placebo sessions included) but no difference in the relative proportions. Underlying data can be found in S1 Data. dE, difference of force levels between the 2 options; dR, difference of reward levels between the 2 options. (TIFF) [file pbio.3000793.s002.tiff]

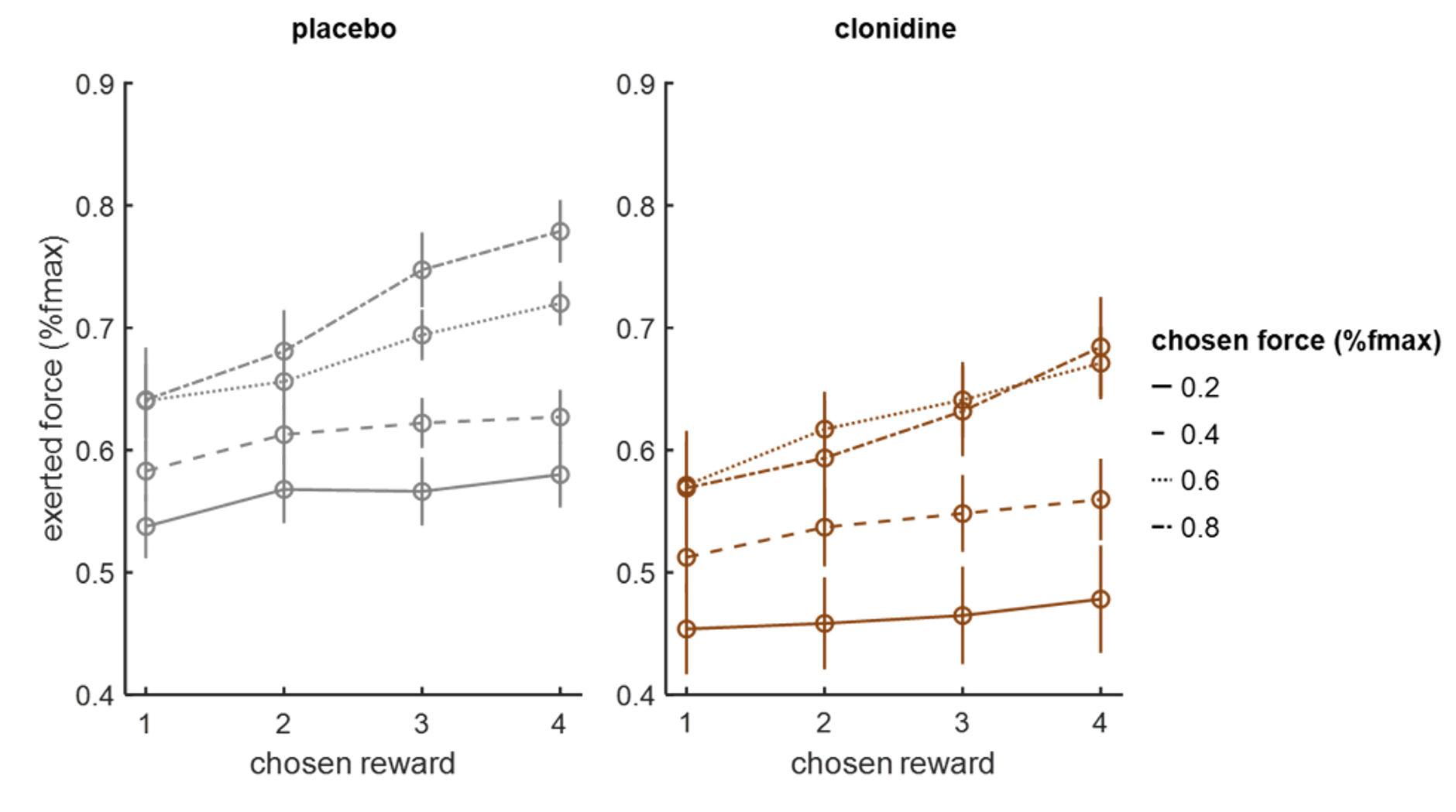

Supplement: S3 Fig — Exerted force depicted as a function of the chosen reward (in abscises) and the required force level (with different line styles), for the placebo (in gray) and the clonidine sessions (in brown). (TIFF) [file pbio.3000793.s003.tiff]
