# Supplementary material for: Pharmacological evidence for the implication of noradrenaline in effort
Source: PLoS Biol. 2020 Oct 12;18(10):e3000793. doi: 10.1371/journal.pbio.3000793 (PMC7580990; doi:10.1371/journal.pbio.3000793)
Supplement: S1 Text — (DOCX) [file pbio.3000793.s005.docx]

*Text S1. Analysis of response-time in the reward-effort choice task*

In the first examination of the behavioral data, we inspected how the response time (RT) of the monkeys related to the experimental conditions. In order to be consistent with the former approach, only behavioral markers demonstrating a consistent effect of the cost and benefit manipulations were kept for further analysis in the drug conditions. Unfortunately, this was not the case in the exploratory analysis that we conducted. In principle, response time during this task aggregated several processes: option valuations, option comparisons and action preparation. We started with the initial assumption that option valuation was the dominant determinant of monkey’s latencies (similarly to the participation rate which usually follow the opposite pattern of response times). Therefore, we tested an effect of the sum of rewards and the sum of efforts offered on response time but neither reached significance. To relax the initial assumption, we considered alternative scenarios for the determination of response time in this task and compared them in a formal model comparison approach. We formulated four possibilities: 1) response-times reflect offer valuation and is impacted by the sum of options values (our initial hypothesis), 2) response-times reflect option comparison before attribute integration and is impacted by the difference of option values, 3) response-times reflect action preparation after the option is selected and is impacted by the chosen option value or 4) response-times reflect option comparison after attribute integration and is impacted by a global choice uncertainty metric. For the last formulation, we chose to represent the choice uncertainty with the entropy of the joint participation and choice probabilities derived from the logistic regressions, which can be understood as the wavering between the three implicit options of the task (participating on the left, right or not participating). The result of the model comparison showed a clear winning model (*freq =* 0.91*, ep* > 0.999), the choice uncertainty model (the last one). This demonstrates a commonality in the determinant of response-time across monkeys and sessions but was insufficient to qualify as a valid behavioral marker of cost-benefit computations. Indeed, the best model doesn’t allow us to identify separately influences of the reward and effort on response-time and the estimation of behavioral parameters depends upon the other pre-defined behavioral markers (participation and choice) which makes this marker redundant and less robust.
