# Supplementary material for: Pharmacological evidence for the implication of noradrenaline in effort
Source: PLoS Biol. 2020 Oct 12;18(10):e3000793. doi: 10.1371/journal.pbio.3000793 (PMC7580990; doi:10.1371/journal.pbio.3000793)
Supplement: Attachment — Submitted filename: Borderies et al_PLOS_V2_resp to reviewers.pdf [file pbio.3000793.s013.pdf]

Dear Dr Gasque,

Thank you for giving us the opportunity to submit a revised draft of my manuscript titled *“Pharmacological evidence for the implication of noradrenaline in effort”* (PBIOLOGY-D-19-02919R1) to *Plos Biology*. We appreciate the time and effort that you and the reviewers have dedicated to providing your valuable feedback on my manuscript. We are grateful to the reviewers for their insightful comments on my paper. We have been able to incorporate changes to reflect most of the suggestions provided by the reviewers. We have highlighted the changes within the manuscript.

Here is a point-by-point response to the reviewers’ comments and concerns.

Reviewer 1

- The description of the behavioral task seems to report data from sessions where vehicle was infused but this should be confirmed. Moreover it is unclear how combinations of choices are presented*. It is really completely random so a high effort/high reward task may be compared to a low effort/high reward option? Were measures taken to ensure that choices differed on both effort and reward?*

The experimental conditions differed between trials in their reward level (1, 2, 3 or 4 water drops) and required force (20, 40, 60, 80% of maximal force), assigned to each option (left or right). These conditions were assigned to a trial number with a random permutation of the 256 different conditions (4 reward levels and 4 required force levels for both left and right options), reinitialized at the end of each blocks of 256 trials and at the beginning of each new session (with an independent permutation).

The reader can find this information in the Method section (page 6).

- If not, do the behavioral data show that monkeys act rationally when choices differed on only one dimension e.g. if reward is equal do they always choose low effort? Or if effort is high for both options do they always choose the larger reward?

Behavioral analyses supported the fact that monkeys were globally rational (in terms of choosing the option with maximum reward and minimal force required) on uni-dimensional choices (choice set with options differing only onto one dimension). Of course, there were a few individual trials where monkeys did commit suboptimal choices, but these were captured quantitatively, either by a systematic bias (laterality or perseveration bias) or by the random error term in the model.

- In general more details about option presentations, randomization and the overall running of the task should be provided. Additionally, the total number of each reward/effort pairing analysed should be reported.

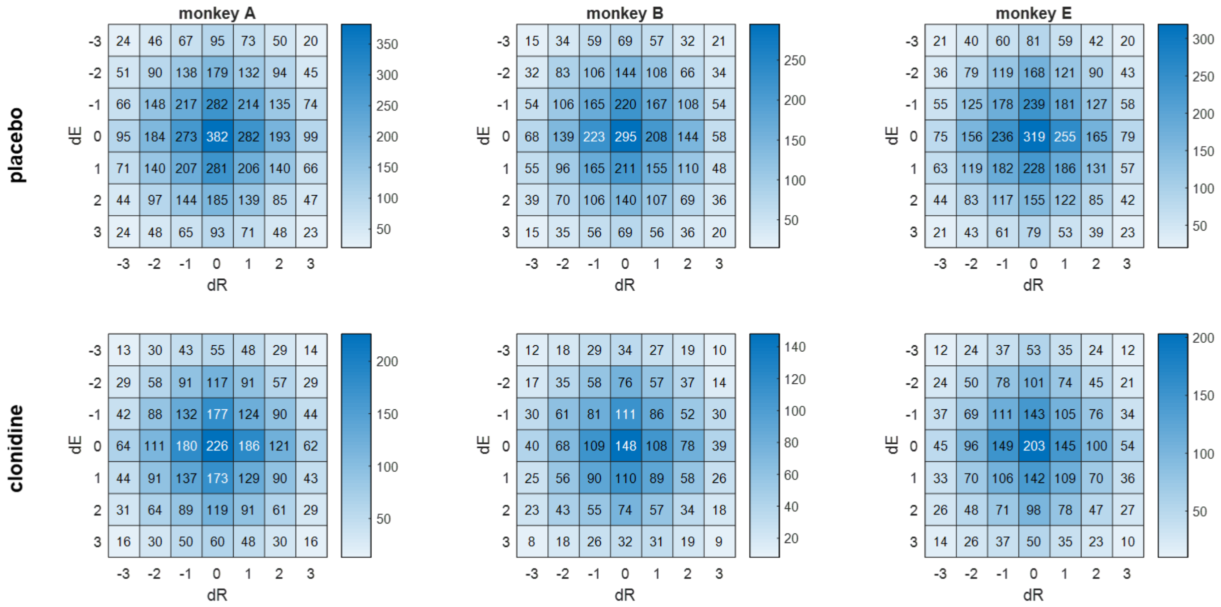

Figure 1. Distribution of experimental conditions across placebo and drug sessions for all monkeys. dR = difference of reward levels between the two options. dE = difference of force levels between the two options. Absolute numbers of trials are indicated inside each box. Note that there is a difference between the placebo and drug session in the absolute numbers (because there were more placebo sessions included) but no difference in the relative proportions.

We checked that experimental factors were similar between placebo and drug session in the statistical distribution of reward differences (dR) and force required differences (dE) for all monkeys (monkey A: chi2(49) = 6.79, p >0.99 ; monkey B: chi2(49) = 10.6 , p >0.99 ; monkey E: chi2(49) = 7.9 , p >0.99). This figure and associated information will be provided in the supplementary material.

➢ Graphs and data analyses are said to generally support the assertion that monkeys understood the task and can discern options based on force or reward. However, it is unclear how subjects behave based on the combination of these cues. For example, what happens to participation on trials when there is a high force/low reward requirement?

Participation to the task was mainly determined by value of both options (the sum of offered rewards having a positive influence, the sum of required force having a negative influence). Thus, monkeys were less inclined to participate when both offered rewards were low and both required forces were high. We used a computational model to further quantify the way monkeys adjusted behavior (participation, choice and force produced) as a function of the combination of effort and reward information. We have extensively revised the description of this model, both in the methods and in the result section, and we hope that it is clearer now.

➢ Figure 2B suggests that monkeys exert roughly the same amount of force for all levels of reward i.e. roughly 60% of max regardless of reward earned. Is the relationship clearer if data from individual monkeys is provided in dashed lines?

We believe that the reviewer was confused about the task, and we made an effort to explain it in greater details. In particular, since this task was intended to study the influence of NA on efforts separately from reward, we did not use the classical ‘incentive motivation’ task where the exerted force is driven by the reward. In such situation, it is almost impossible to dissociate effort from reward. Here, since effort and reward were manipulated independently, we could show that clonidine only affected effort (chosen force difficulty and amount of force exerted) but not reward-related processes (reward based choices and

reward-related exerted force). The group and individual data illustrate well that the main driver of exerted force is the required force (since it is instrumental in this task) and the incentive effect of chosen reward on exerted force (not instrumental in this task) was relatively limited. We will add this figure in the supplementary material.

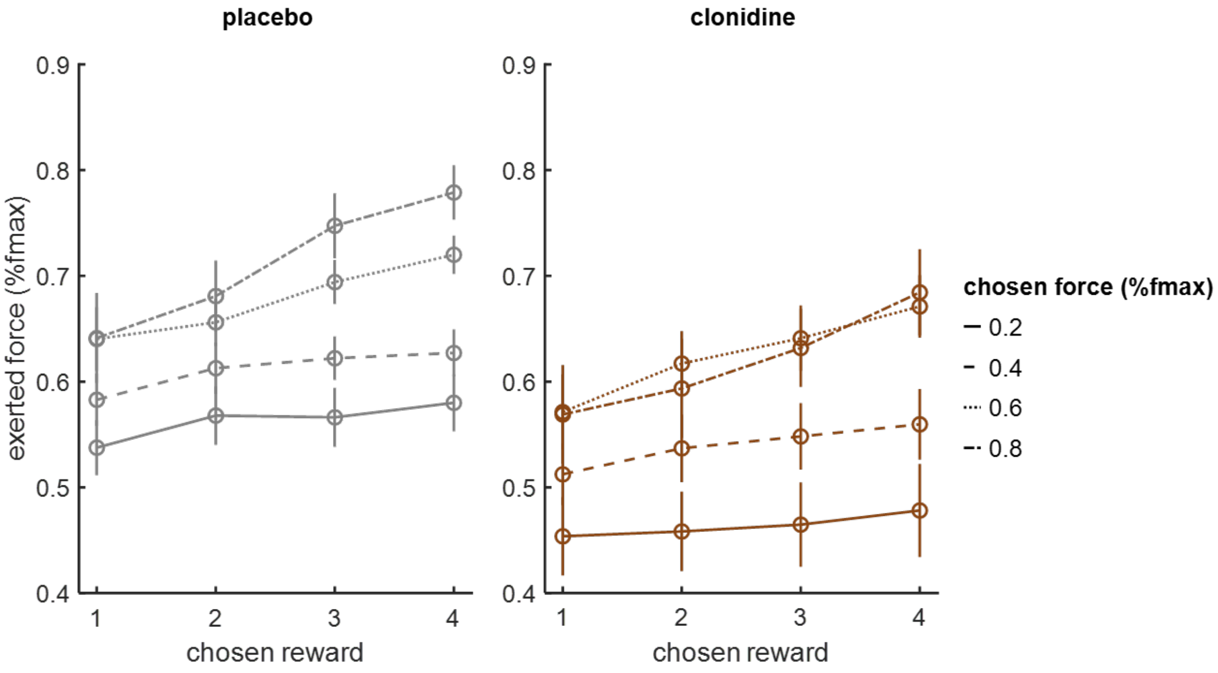

Figure 2. Exerted force depicted as a function of the chosen reward (in abscises) and the required force level (with different line styles), for the placebo (in grey) and the clonidine sessions (in brown).

➢ The concern about the lack of variation in force exerted is not alleviated by the data shown in Figure 2A which shows monkeys exert much more force than need for the lowest two levels of force assessed suggesting they have difficulty titrating force exerted.

The reviewer raised an important point here. Monkeys did overshoot for low required force levels and undershot for high force levels, meaning they had difficulty to control their exerted force (as it is the case for humans in ballistic force contractions, see Oudiette, et al. 2019) despite the fact that they followed the instruction (as demonstrated by the fact that their exerted force depended strongly on the required force). We believe this is not a concern for the interpretation of task results: effort cost as (measured by choices) does follow a monotonic and positive relation with required force, and this cannot be interpreted in terms of risk. Altogether, we believe that our data are compatible with the idea that monkeys did understand the task, even if they had some difficulty to execute some of the required forces with enough precision.

➢ These same concerns limit interpretation of the clonidine data. One point to consider if the authors want to argue against clonidine impacting vigilance is to analyse standard metrics of vigilance e.g. level of vigilance assessed in the first few minutes of the task or to assess changes across the testing session.

A simple regression analysis of response time could not retrieve any significant effect of clonidine, neither on response time at the beginning of the session (RT(placebo)= 0.505 ± 0.087 s.; RT(clonidine)= 0.539 ±

0.069 s.; $F(1,145) = 1.366$, $p = 0.244$) nor on linear variations across the session ($\Delta RT/\Delta time(placebo) = 0.045 \pm 0.042$ s. ; $\Delta RT/\Delta time$ (clonidine)= -0.011 ± 0.031 s.; $F(1,145) = 1.385$, $p = 0.241$). This does not elude the possibility that clonidine affected vigilance of monkeys because the trend is toward a slowing down of monkey responses. This has been added to the result section (p. 10). However, it could totally be a motivational effect that impact decision uncertainty, a major factor in the determination of response time (see supplements).

- ➢ Check in text references to Figures 2D and 2E in results section as they seem to be switched.

Thank you for the typographic mistake. This has been corrected.

Reviewer 2

Introduction

- ➢ Given the central importance to the paper, I felt that the exact definition of effort used could still be explained more clearly. Undoubtedly the authors have tried to do this, but it would not hurt to make the definition even more explicit, and also discuss potential drawbacks of the definition (i.e. the assumption that the weighing of effort at time of choice is strongly related to the subsequent production of force). In fact, it could be argued that one downside of the experimental paradigm is that it does not completely dissociate these factors (as an agent's choice is signalled by the required force production) which could in theory contribute to the observed results converging on the common variable of 'effort'.

We thank the reviewer for this constructive remark. We made significant changes in the introduction, to clarify both our operational definition of effort and our general position vis a vis effort. In short, our objective here is clearly not to provide a theoretical definition of effort, or to challenge any of the existing definitions of effort in the literature. Rather, we focus on isolating effort from potential confounding factors, including reward and incentive motivation. Indeed, many experiments on effort use incentive task where the amount of reward earned scales with the amount of force exerted, such that it is extremely difficult to dissociate effort from reward or force. Here, we wanted to avoid this difficulty by manipulating force and reward independently, such that effort could readily be dissociated from reward and force. Again, our intention is to to get into a theoretical debate on what effort is or should be. Rather, we wanted to test the hypothesis that besides the well known, DA dependent, incentive effect of reward on behavior, there could be a separate system system influencing energy mobilization and its influence on choice and action. Although we clearly do not think that our study provides a definitive description of what effort is, from a theoretical and biological point of view, we believe that it will help the field to move forward. Finally, even if the same behavioral response (squeezing the bar) reflects both decision and force production, it is still possible to dissociate the two because monkeys do not necessarily exert the amount of force chosen. Again, our goal is is not to demonstrate the effort is exactly the operational variable that we used here, but we believe that it is important to demonstrate that a noradrenaline dependent variable can influence both decision making and force production, independently from reward.

- ➢ The authors discuss the more tenuous relationship between dopamine and effort, citing many relevant articles. However, human studies assessing choice behaviour with dopaminergic manipulations have demonstrated changes in both physical effort (Le Heron 2018 Brain) and cognitive effort (Chong 2019 Brain) suggesting the conclusion of this paragraph ("…its implication in effort remains limited when effort is dissociated from reward and force.") should be tempered. Furthermore, the former, along with Zenon 2016 and Le Bouc 2016 (cited already) do demonstrate seemingly invigorating effects of dopamine on motor performance (defined in varying ways). Finally, although clearly important to consider dissociable systems underlying effort and reward processing, it should also be remembered that effort as a construct may not exist without some sort of goal, or reinforcer (effort is targeted), and thus at a higher level may not be strictly speaking dissociable from reward.

Again, we thank the reviewer for this remark, this is indeed a critical issue. As explained just above, our intention was not to provide a definitive definition of effort or contradict the enormous literature showing

the incentive effect of DA on effort, if effort is defined as mobilization of energy for action and/ or cognition. We have added a sentence in the introduction to clarify that point, and we included these key references. Our intention was to evaluate the possibility that when energy mobilization can be dissociated from reward, at least in the short run/ at a low level of abstraction, it could involve another system (noradrenaline). We agree with the reviewer that, from a theoretical point of view and at an infinite time scale, energy expenditure (effort?) is targeted (energy is invested to reach a goal). We may assume that when monkeys choose to exert a high level of force over and above the incentive effect of reward they « make an effort » to reach a distant goal, such that the reward could be defined as « being allowed to perform the next trial, which could be more beneficial ». From that perspective, the level of representation and the time course of the incentive effect of reward (or its very nature) becomes just as complicated as that of effort. But we are not willing to speculate in that direction, because as exciting as this theoretical discussion on effort could be, it is way beyond the scope of our work. Our only claim here is that when animals need to mobilize energy to face an immediate difficulty, and when they cannot rely upon the immediate incentive effect of reward to do so, they can rely upon noradrenaline. And we hope that it will help understand what effort really is about. We have rewritten the final conclusion of the paper to clarify this.

Methods
Task

- It is obviously not a straightforward issue to estimate maximal force for each monkey, whilst being important – the method used seems like a good solution – were there situations during testing where the monkeys exceeded the 'maximal' force?

The maximal overshoot was 151% of the calibration force. Average empirical maximal forces were 102.61 ± 1.25 % (mean ± sem) meaning that calibration were close to the empirical capacities of the monkeys. This has been added to the methods section (p.5).

- How was the 4x4 option space sampled across the expt? This is important in terms of interpreting the raw and modelled data – did all monkeys on all sessions receive approximately the same range of options, or were there any systematic differences?

This is a similar response to Reviewer 1 concern.

The experimental conditions differed between trials in their reward level (1, 2, 3 or 4 water drops) and required force (20, 40, 60, 80% of maximal force), assigned to each option (left or right). These conditions were assigned to a trial number with a random permutation of the 256 different conditions (4 reward levels and 4 required force levels for both left and right options), reinitialized at the end of each blocks of 256 trials and at the beginning of each new session (with an independent permutation).

The reader can find this information in the Method section (page 6).

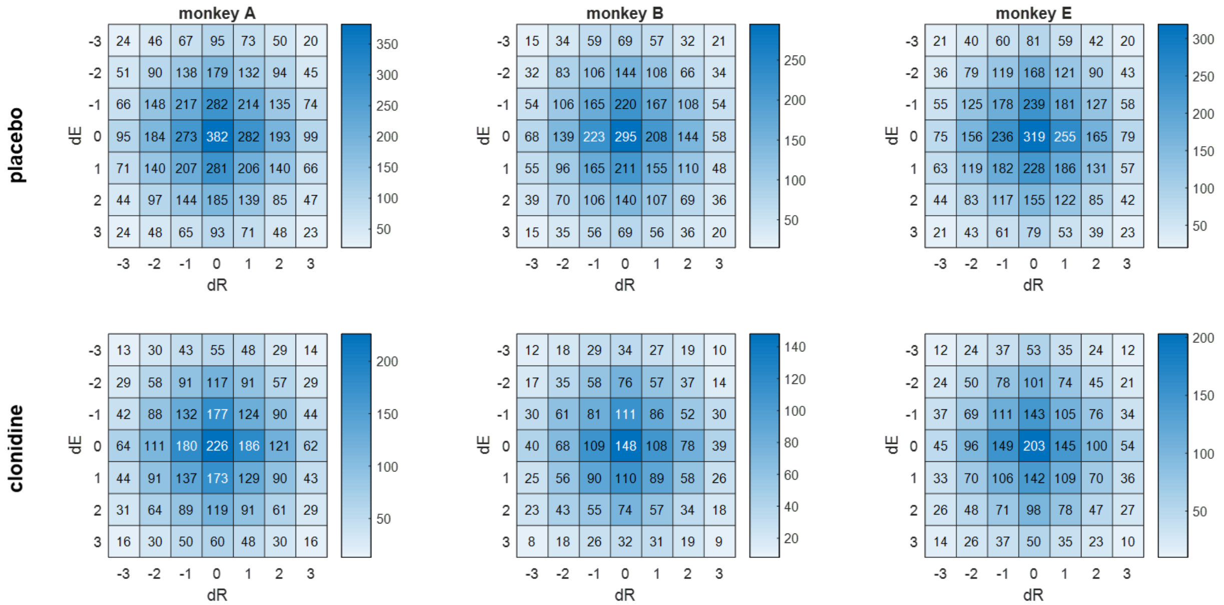

Figure 1. Distribution of experimental conditions across placebo and drug sessions for all monkeys. dR = difference of reward levels between the two options. dE = difference of force levels between the two options. Absolute numbers of trials are indicated inside each box. Note that there is a difference between the placebo and drug session in the absolute numbers (because there were more placebo sessions included) but no difference in the relative proportions.

We checked that experimental factors were similar between placebo and drug session in the statistical distribution of reward differences (dR) and force required differences (dE) for all monkeys (monkey A: chi2(49) = 6.79, p >0.99 ; monkey B: chi2(49) = 10.6 , p >0.99 ; monkey E: chi2(49) = 7.9 , p >0.99).

➢ Is force production really dissociated from choice? Monkeys signal there preference by producing force for the chosen option – an accept decision therefore suggests they are prepared to exert the required force.

This is a good remark. The decision to participate to a trial and force execution were expressed by the same physical response (squeezing one of the two handgrip). This mean that there is no temporal dissociation between the two behavioral measures in this task. We believe this is not problematic since we do not claim that these two measurements are two fundamentally distinct processes. Even from a theoretical perspective, there is probably some overlap between 'choice', 'action preparation' and 'action execution', such that dissociating these 3 functions might be just as artificial as associating them. In a context of animal work where monkeys are trained for months to execute the same task, we feel that it was important to take advantage of their enormous practice with the task, which allows them to build a reliable representation of the required force when given the cue and required to prepare the action. Moreover, even if in terms of timing 'choice' and 'force exertion' occur at about the same time, they can be dissociated across trials since monkeys do not systematically express the amount of force required by the chosen difficulty level (required force). Critically, the effects of noradrenaline could have been restricted to only one of these two measures (choice vs amount of force exerted). Thus, the strong correlation (across sessions) between pharmacological effect on choices and force produced does reinforces this idea that noradrenaline affects a single process (which we call effort), which affects both choices and force production.

➢ Was there any physiological data (e.g. HR, pupil diameter etc) to support the presumption that Clonidine was in fact reducing NAd levels?

That would have been a valuable information but this was not recorded for technical simplicity. These autonomic measures are so indirectly related to the LC that we did not feel that the benefits (relative to indirect evidence based on the literature) was not worth the cost (more training and physical constraints for the monkeys).

Results:

- ➢ Although the authors attempted to mitigate for this potential effect, the fact that monkeys on average appeared to produce a force quite substantially less than required for high force options (Fig 5C) is concerning, and, at a behavioural level would still seem a possible explanation for the observed interaction in Fig5A – the flat line between 60 and 80% of max force suggests (on average) that monkeys had reached their max force production at the 3rd force level.. Do the authors have any data to suggest that maximal possible contraction was actually the same (or different) ON and OFF Clonidine (e.g. maximum squeeze achieved throughout each session in each condition).

Exerted force was not significantly higher for the fourth required force level compared to the third force level overall (beta(level4>level3) = 0.0285 , t(212) = 1.49 , p = 1.38e-01) but the trend is toward a positive effect (meaning that monkey did not exert less force for the fourth force level). Moreover, the same comparison between fourth and third force level was significant when we considered only placebo sessions (beta(level4>level3) = 0.0438, t(138) = 3.04 , p = 2.87e-03) and not significant when we considered only clonidine sessions (beta(level4>level3) = 0.00319 , t(72) = 0.0946 , p = 9.25e-01).

Furthermore, the maximal force recorded within experimental sessions were significantly reduced under clonidine compared to placebo sessions (empirical Fmax(placebo)= 94.42 ± 3.86% ; empirical Fmax(clonidine)= 105.75 ± 1.91% ; F(1,145) = 10.4 , p = 1.55e-03). Thus, we cannot rule out the possibility that clonidine affected maximal force power but a motivational effect of clonidine would also predict an effect on maximal empirical forces (as illustrated by model predictions). This information has been added to the result section (p. 8). We had conducted a set of separate control analysis to confirm that the effect of clonidine could not be reduced to a simple motor impairment, and we have edited that section to clarify that point (cf p.10).

- ➢ Although the results are specific for an effort component, it remains possible that the effect of clonidine could have occurred via some other mechanism (for example a peripheral change in muscle contractility). If the animal "knew" it was weaker ON Clonidine then the observed reduction in high effort choice could be a natural corollary of this – it was simply adjusting to its new motor state. These issues should be either addressed, or at least discussed in more detail in interpreting results.

We acknowledge that this is a limitation of our study that we addressed with control analyses on force kinematic parameters (Figure 6) and emphasized this more in our discussion.

- ➢ Could the relationship between Reward and force exerted simply be explained by animals being more likely to accept high force offers when reward was higher? A more interesting presentation of these results might be to see if reward on offer modulated exerted force at each required force level.

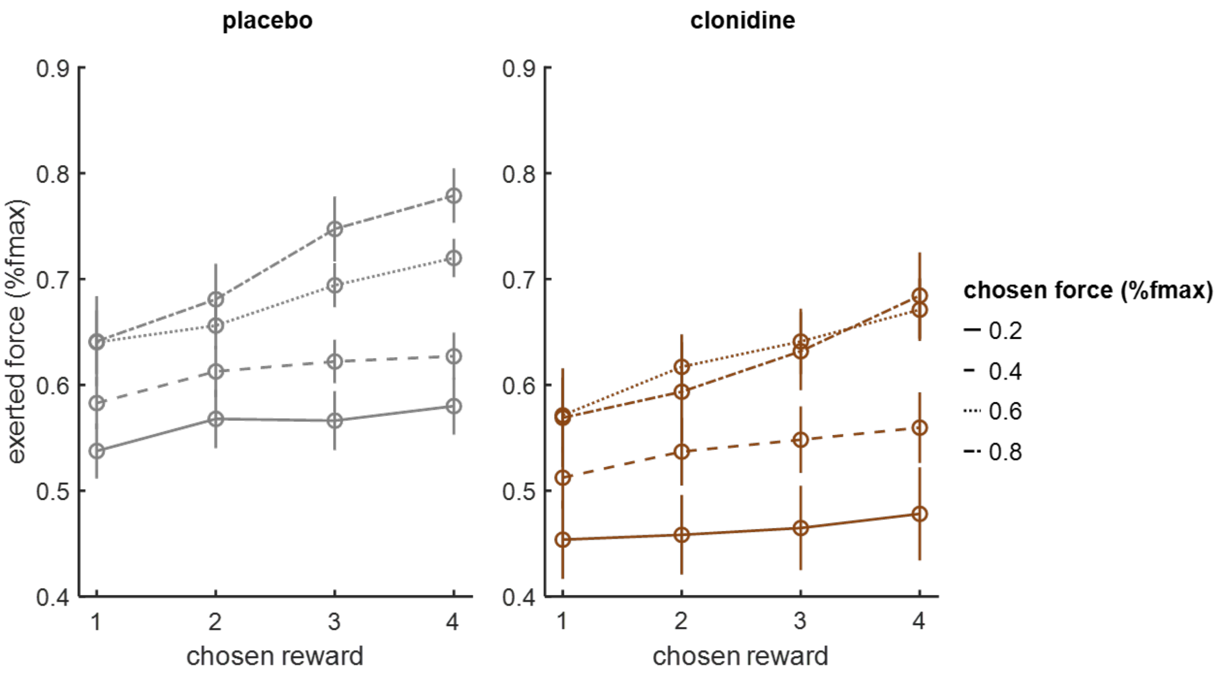

Chosen reward had a positive effect on force exertion, as indicated by the results of the GLM regression (beta_reward=0.02, t(147)= 3.205, p=0.001), which was independent from a direct effect of chosen force level or an interaction between force level and chosen reward. This relationship was still significant (beta_reward=0.02, t(147)= 3.0499 , p=0.002) even if we remove the variance explained by the required force level in a first step and perform the regression analysis of chosen reward and force exerted in a second step (shortcutting the assumption of linearity embedded in the GLM analysis). This information has been added to the supplementary material.

Discussion

- ➢ Given the time spent in the introduction discussing the relationship between effort-based choice/ motivated behaviour and dopamine, it would be helpful for the authors to return to this issue, and discuss how their findings regarding the Noradrenergic neuromodulatory system fit into the broader picture of motivated behaviour – particularly because, as they mention, dopamine still has a clear role in activating behaviour towards goals (appetitive behaviour).

We definitely agree with the reviewer, we hope that the changes discussed above (ie in the introduction and the final conclusion) will partly address this issue. We have also added a sentence in the discussion (section ‘dual impact of clonidine…’, first paragraph.

.

- ➢ The authors briefly mention in the introduction the potential importance of neuromodulatory systems for clinical problems – it may be worth returning to this in the discussion, as these findings may provide an important neurobiological addition to recent frameworks for problems such as apathy (e.g. Le Heron 2019 JNNP) and Fatigue (e.g. Kuppuswamy 2017 Brain).

We have added a paragraph on clinical applications at the end of the discussion.

- ➢ Atomoxetine is mentioned under drugs – but is not referred to in the introduction or the rest of the manuscript…

Thank you for the typographic mistake. This has been corrected.

- ➢ Why was the time to assess the option jittered? In a purely behaviour task this doesn't seem to add much, and possibly may have confused the monkeys e.g. leading to early responses?

This is a standard practice in the field. This procedures enhances the tendency of animals to respond to (jittered) stimuli, rather than ignore them and respond after a fixed time. The major source of confusion would actually be a fixed time, because time itself would become a cue and competes with the visual stimulus, inducing early responses. We are not aware of situations where fixed times are better than variable times, and if anything, this procedure makes the comparison with neurophysiological and neuroimaging experiments easier.

Model:

- ➢ The authors could perhaps justify their choice of model variables (e.g. quadratic effort, sigmoid reward) – these are reasonable choices but should be backed up by the literature.

We included relevant references in the manuscript to justify these modeling choices (e.g. methods section, page 9).

- ➢ Fig 2A – although animals modulated force to requirements, they clearly significantly oversqueezed on low force options – is this problematic at all in terms of analysis?

Over-squeezing for low required forces, or baseline force, should not cause any bias in the statistical analyses. Standard model-free analyses captured the effect of baseline force with the intercept parameter of GLMs. Model-based analyses captured the effect of baseline force with the K0 parameter of the model. However, it should be noticed that these two metrics are not strictly identical because the K0 parameter take is fitted with multi-source data (participation and choice data in addition to force data) and is influenced by non-linear effects of other parameters capturing the patterns in force data. This illustrate why clonidine can affect the intercept parameter in model-free analyses and not the K0 parameter in model-based analyses.

Reviewer 3

- ➢ My issues mostly have to do with the detailed computational model. The model is not well-described in the main text. The reader should not have to go to the supplementary material to try to decipher the model.

We tried to clarify the description and justification of the computational model in the method section while keeping it concise. We believe two levels of reading are useful to present the methodology of computational models. The main text was written so that the reader can grab the logic and intuitions of the model. The supplemental material was written to assist the reader that want to go further in understanding the details of the equations.

Here are some examples:
1) what is N?;

N refers to trial number. It is is explained in the main text.

2) what is F*;

F* is the optimal force to be exerted on the grip. We added some explanation for this passage which was opaque.

3) what is Eoption?;

Eoption is the required force for this option. We have added an explanation in the methods and in the supplemental material.

4) Throughout there is the use of the term expected force and required force - what are these?;

There was actually a typo, ‘expected force’ should have been ‘exerted force’ (as on the figure). This has been corrected. The exerted force is the amount of force actually exerted on the grip, whereas the required force is the level of difficulty, the amount of force that monkeys must exert on the grip to complete the trial. We have clarified this both in the methods and in the supplements.

5) the equation e[F] = ... is non-sensical: the lhs is a number (the expected value of an (undefined) random varible) and the rhs is a random variable.

This is correct, the expectation of F is the optimal force of the value function, the prediction of F is the optimal force of the value function plus an error term. We adjusted the equation so that the reader does not get confused.

If the model is introduced in the main text including the basic equations - everything in them needs to be defined and well-motivated. With this exposition in its current state it is not possible to evaluate the calm related to this model.

- I also have a broader concern. The authors claim at various times that "effort" has been defined. I don't believe it has been. There are some fairly complex sentences about how it has an impact on this and that, but that is circular! ("In short, effort is defined as a single computational variable having a dual influence on behavior: a negative influence on choices (all other things being equal, monkeys minimize energy expenditure) and a positive influence on force production (monkeys exert more force and therefore spend more energy for high-force options"). I do not see effort in the equations... This makes understanding the ms and evaluating the results not possible.

Thank you for this remark. This is a critical issue, raised by the other reviewers. We have rewritten the introduction and discussion to clarify what we meant by ‘effort’, and more generally speaking what was he aim of the study. The goal of this study is clearly not to provide a strong theoretical definition of effort in general, and to confront this definition of effort with others in the literature. Rather, we intend to study the role of noradrenaline in ressources mobilization for action independently from reward (and thus based on expected cost rather than expected benefits.). This has been clarified in the 2nd and 3rd paragraph of the introduction.

We look forward to hearing from you in due time regarding our submission and to respond to any further questions and comments you may have.

Sincerely,

S. Bouret
