# Supplementary material for: Pharmacological evidence for the implication of noradrenaline in effort
Source: PLoS Biol. 2020 Oct 12;18(10):e3000793. doi: 10.1371/journal.pbio.3000793 (PMC7580990; doi:10.1371/journal.pbio.3000793)
Supplement: Attachment — Submitted filename: Borderies et al_response to reviewer4.pdf [file pbio.3000793.s015.pdf]

Dear Gabriel,

We would like to thank all reviewers for their time and for their globally positive evaluation of our work. Please find below our specific responses to reviewer 4.

Best regards,
Sebastien.

> Reviewer #4: Borderies et al., present a neuropharmacology study in monkeys, aimed assessing the causal role of noradrenaline in effort-based decisions and the exertion of force. Using a physical effort-based task and the pharmacological agent clonidine, they show that reducing noradrenaline levels reduced the willingness to choose to exert effort, and exertion of force.
Overall, this an is interesting study, that tests a well-justified hypothesis, that noradrenaline levels are linked to effort processing. Although the results are not particularly surprising or a major advance given the labs existing data (Varazzani et al., 2015, JoN), and at times the manuscript could be much more clearly written, it is neat to have a causal demonstration of a link between noradrenaline and effort. Overall, the authors have done a good job of addressing the existing reviewer's comments and I will not add significantly to the burden of the authors by making many additional points. However, I do have some comments that I think warrant being addressed.

***We thank the reviewer for this globally positive evaluation of our work.***

> Major

> 1. In the revisions the authors argue that the results could not be explained by risk, despite the subjects showing an undershoot in force exerted at the higher levels. However, I am not entirely convinced by the response, as from my reading it cannot be determined whether there is a contribution of risk to the effort discounting process in this work. I think that this should be explicitely stated as a limitation in the Discussion, no matter whether the authors believe it unlikely.

***We thank the reviewer for this remark. We have edited the discussion to add risk as a potential confounding factor.***

> 2. I like the evidence presented that effort based choice and effort production could be unified. But, I wondered how much this is down the paradigm, compared to how often it is that effort and reward become linked together. This was a point raised by other reviewers, that I did not feel was adequately addressed. The reason I believe it is important as there is lots of evidence that at a more abstract level, effort and reward can become contingent on each other (Pooresmaili et al., 2015, PNAS), effort can sometimes become valued (Inzlicht et al., 2019, TICS) and sometimes there are contexts when choosing to exert effort does not map on to being willing to exert as much force - such as in the social condition of Lockwood et al., 2017, Nature Human Behaviour. Again, I think the authors should more simply state in the discussion that this experiment is an example where effort and reward can be dissociated, but such a dissociation depends on the context, with many situations where effort and reward become linked or choice and force production become unlinked, which may complicate the simple story of noradrenaline increasing motivation to exert effort.

***We thank the reviewer for this remark. We have edited the discussion accordingly. More specifically, we acknowledge that this is an artificial task which aims at dissociating effort and reward to study their respective neural basis and limit the confounding interpretations. But clearly, energy intake (rewards) and expenditure (effort) must be balanced in the wild, so ultimately they are strongly interdependent. We also acknowledge that the relation between effort and reward can become very elaborate and multi-faceted constructs in humans, and therefore display non-trivial properties that are way beyond the scope of that study. In that respect the conclusions of our study should indeed be taken with caution and not overgeneralized.***

> 3. Several of the reviewers noted that the definition of effort was not clear. The authors have attempted to clarify, this in paragraph 2 and 3 of the introduction. However, I still found the definition hard to follow.

There are several grammatical mistakes in the paragraph making it more challenging. Would they not be better off saying that there is (i) The objective difficulty of a task, (ii) the degree to which a subject is willing to choose to exert the effort required to complete that level of task difficulty and (iii) the energisation of the action, and thus exertion of effort, to complete the task. Following that the aim of this study was to design an experiment that dissociated effort and reward in both decision-making and force production. This would seem simpler than the current lengthy description and definition in those paragraphs.

***We respectfully disagree with the reviewer for 3 reasons: 1) his/ her alternative definition appears like a list of items rather than a definition per se. From that statement, it is not clear how these 3 items (the objective difficulty of the task, the degree to which a monkey is willing to perform a given action and the energization of the action) are supposed to relate to each other and to effort in general. 2) Given the definition of the reviewer, it is not clear how effort and exerted force differ. 3) Most importantly, our work aims at showing that noradrenaline mediates the action of a single theoretical entity, which we refer to as effort and capture using computational modeling. Thus, we believe that it is essential to define effort as a single variable that can affect both choices and force production. As mentioned by the reviewer, our definition of effort was not clear in the initial version of the manuscript but the initial reviewers seemed satisfied by the updated definition, which was rewritten based on their recommendations. We are therefore sorry to decline the reviewers suggestion to change that definition.***

> 4. Changes across trials - A previous reviewer raised the concern about changes in vigour over trials. Although the authors suggest there is a trend, but it is not a major concern, I do think it would be better to say this explicitlyis a limitation and could be addressed in future studies in the discussion. There are of course studies showing that fatigue impacts on the willingness to exert effort for reward (Meyniel et al., (2013); Meyniel et al., 2016; reviewed in Muller & Apps, 2019). I don't think the task is set up to examine these effects appropriately, and this should be noted as a limitation in the discussion.

***We thank the reviewer for this remark. We have edited the discussion accordingly. More specifically, we acknowledge that fatigue could account for slow changes in behavior across trials of a session, and cite one of the mentioned references. We also acknowledge that the study was not meant to study fatigue and that this can be taken as a limitation.***

> 5. In the abstract it refers to a "single hidden variable" as one of the key results. However, it is not until the manuscript has been read that it is clear what this actually means. This makes understanding the abstract very difficult. The authors need to clarify this result in the abstract much more clearly.

***We thank the reviewer for this remark. We have expended the explanation of effort as a single hidden variable in the abstract. We hope that it is clear enough now.***

> MINOR

> 6. The manuscript state that the model was similar to the quadratic model of klein-Flugge et al. (2015) and Hartmann et al., (2013). However, the model in Klen-Flugge et al., was in fact a sigmoidal model, whereas in Hartmann et al., (2013) the model was parabolic. The authors should be more clear in the description here of whether it is a sigmoisal or parabomic model, as has been used more extensively in the literature (see Chong et al., 2017, PLoS Bio).

***We thank the reviewer for pointing this important detail. Indeed, Klein-Flugge et al. (2015) study effort discounting using a sigmoidal model (winning clearly against a hyperbolic model) but based on the information provided in the supplemental methods (Fig S2), the sigmoidal model is not significantly better (in terms of excedance probability) than the other parabolic models (quadratic, power function). Moreover, the authors stated clearly that their design was not made to distinguish different concave shapes for the discounting function, but to distinguish families of models with concave functions (e.g. quadratic) for effort discounting from models with convex function (e.g. a hyperbolic) for delay discounting. In that respect, the reference provided by the reviewer (Chong et al.( 2017, PLoS Bio), is indeed providing a more straightforward evidence and we have included it in the corresponding section.***

> 7. It would help the ease of understanding of the paper, if the if Kr, Ke etc were defined in every figure legend where they are included. For instance, in figure 4 they aren't defined, which adds to the burden for the reader.

***We have added a definitions of these key variables in the legend of figure 4***

> 8. There are many grammatical errors throughout the manuscript. As there are no line numbers it became difficult to note these down. However, I would suggest a native speaker is given the manuscript to read through, to correct the many errors in the text.

***We appreciate the suggestion, and hope to work with the journal to make sure that the quality of the manuscript is up to their standard.***
